# Increased carotid intima–media thickening and antioxidized low-density lipoprotein in an anti-Ro60 SLE autoantibody subset

**DOI:** 10.3389/flupu.2023.1197309

**Published:** 2023-09-26

**Authors:** Biji T. Kurien, James Fesmire, Swapan K. Nath, R. Hal Scofield

**Affiliations:** 1 Arthritis & Clinical Immunology Program, Oklahoma Medical Research Foundation, Oklahoma City, OK, United States; 2 Department of Veterans Affairs Medical Center, Oklahoma City, OK, United States; 3 Department of Medicine, University of Oklahoma Health Sciences Center, Oklahoma City, OK, United States

**Keywords:** oxidized LDL, antioxLDL, atherosclerosis, plaque, SLE, autoantibody subset, carotid intima-media thickening

## Abstract

**Objective:**

Premature atherosclerosis is associated with systemic lupus erythematosus (SLE). We have previously shown an association of anti-Ro60/La/Ro52 with antioxidized low-density lipoprotein (LDL) in SLE. Here, we hypothesized that carotid intima–media thickening (CIMT) would be associated with antioxidized LDL (anti-oxLDL)/antilipoprotein lipase (ALPL) in a specific SLE autoantibody subset (anti-Ro60 positive, anti-RNP positive, anti-SmRNP positive, or extractable nuclear antigen antibody negative).

**Methods:**

We carried out a case-control study (one time-point testing) of CIMT, ALPL, anti-oxLDL, anti-low density lipoprotein (ALDL), and anti-LDL in 114 SLE patients and 117 age/sex-matched controls. The levels of total cholesterol, LDL, high-density lipoprotein (HDL), triglycerides, and HDL-Trig were also measured. A student’s *t*-test was used for statistical analysis.

**Results:**

Interestingly, the level of CIMT was highest in the SLE subset with anti-Ro60 (23/114). CIMT and anti-oxLDL were statistically significantly elevated in the anti-Ro60 SLE subset (1.3 ± 1.66, *p* < 0.01; 0.26 ± 0.16, *p* < 0.002, respectively) compared with controls (0.54 ± 1.26; 0.165 ± 0.13, respectively), but not anti-LPL/anti-LDL. CIMT was significantly elevated (0.9 ± 1.71; *p* < 0.05) in the SLE subset without antiextractable nuclear antigen (ENA) (63/114) compared with controls. The other antibodies in this subset were not statistically different from other SLE subsets or controls. Only antioxLDL was significantly elevated (0.29 ± 0.27; *p* < 0.005) in the SLE subset with anti-RNP (14/114) compared with controls, while none were elevated in the anti-SmRNP subset (6/114). We did not find any significant differences in lipids between the various SLE subsets.

**Conclusion:**

CIMT segregates in anti-Ro and ENA negative groups either with or without anti-oxLDL. It will be clinically important if cardiovascular events are augmented in the SLE anti-Ro subset having elevated antioxidized LDL antibodies.

## Introduction

Premature atherosclerosis is an important late complication of systemic lupus erythematosus (SLE), while also being an important issue in patients with early SLE. There is an increased prevalence of atherosclerotic plaque formation in SLE subjects, in addition to an elevated risk of cardiovascular disease (CVD) (1–6). Non-traditional risk factors such as cytokines, chemokines, and autoantibodies, in addition to traditional risk factors, contribute to the development of CVD (7–9). Of late, it has become evident that atherosclerosis and its corollary, CVD, are inflammatory ailments and that the immune system influences the development of disease (10). It is, therefore, of interest to investigate the elevated risk of CVD in SLE since immune mechanisms in human atherosclerosis could be elucidated. Autoantibodies targeting oxidized low-density lipoprotein (LDL), cardiolipin, and β2 glycoprotein 1 are associated with SLE and antiphospholipid syndrome–related vasculopathies. Immunochemical epitopes found on oxidized LDL are seen in atherosclerotic lesions (11–13). Antibodies to oxidized LDL are increased in atherosclerosis and are also found in SLE, diabetes, hypertension, and pre-eclampsia (11, 12, 14, 15).

Autoantibodies in SLE target a 60,000 molecular-weight protein (Ro60 or SS-A) or a 48,000 molecular-weight La (SSB) autoantigen of the Ro RNP particle, associated non-covalently with one or more of four short uridine-rich human cytoplasmic RNAs (hY RNAs). Up to 50% of SLE subjects have anti-Ro60. Anti-La occurs in considerably fewer SLE subjects. SLE autoantibodies also target the autoantigen Ro52 (also known as TRIM21) (16–18).

Sm and nuclear ribonucleoprotein (nRNP) antigens are also commonly targeted in SLE. These proteins are involved in the splicing of pre-mRNA in association with U small nuclear RNAs. Anti-Sm autoantibodies are found in the sera of roughly 20%–25% of all SLE subjects. These antibodies form part of the criteria for SLE classification and are highly specific for SLE (19).

It is important to note that mortality from lupus manifestations has diminished due to better treatment modalities. However, deaths caused by CVD from atherosclerosis in SLE have not. In fact, CVD is responsible for more than one-third of all deaths in SLE subjects (20, 21).

Carotid intima–media thickening (CIMT), measured by an ultrasound of the carotid arteries, is a valuable predictor of CVD and is associated with the clinical risk of angina and myocardial infarction (22). Ultrasound has been of great use in detecting atherosclerotic plaque and also in measuring carotid artery intima–media thickness (IMT) (23). Owing to ease of visualization and reproducibility, IMT is preferably measured in the common carotid artery. Furthermore, internal carotid artery measurement has been successfully used to detect and measure carotid plaque in subjects with atherosclerosis/cardiovascular-related conditions (24, 25).

Previously, we investigated the extent of coronary risk caused by antilipoprotein lipase and antioxidized LDL in the context of CIMT in SLE subjects and normal controls (15). The study found that antilipoprotein lipase (anti-LPL) was associated with oxidatively modified LDL, production of antioxidized LDL antibodies, CIMT, and coronary risk in some SLE patients (15, 26).

Based on our recent findings, which demonstrated an elevated susceptibility of SLE subjects with anti-Ro 60, La, and Ro 52 antibodies to develop antioxidized LDL (ox-LDL) antibodies (26), we formulated the hypothesis that atherosclerotic plaque would be associated with antioxidized LDL (anti-oxLDL)/antilipoprotein lipase (ALPL) in a specific SLE autoantibody subset (anti-Ro60 positive, anti-RNP positive, anti-SmRNP positive, or extractable nuclear antigen antibody negative).

## Subjects and methods

### Subjects

Data collected from an earlier study (15) were analyzed after obtaining study approval from the Institutional Review Board of the Oklahoma Medical Research Foundation. This study used 114 SLE subjects (104 women and 10 men) and 117 age/sex-matched controls. None of the subjects had Sjögren’s syndrome. The subjects were not on any lipid-lowering medication. The study subjects met the SLE 1982 revised classification criteria of the American College of Rheumatology. The OMRF Clinical Immunology Laboratory, a CLIA-approved facility, carried out serological studies. Antinuclear antibody (ANA) was tested by indirect immunofluorescence using a HEp-2 substrate. Antidouble-stranded DNA was determined by *Crithidia lucilliae* immunofluorescence and autoantibodies to extractable nuclear antigens by double immunodiffusion. Normal controls were selected to match the SLE subjects for sex and age. None of the controls were taking any lipid-lowering medications. The study was approved by the Oklahoma Medical Research Foundation Institutional Review Board.

## Methods

All assays were carried out in the previous study and reported (15) and are briefly described here.

### Carotid bilateral ultrasound

The CIMT procedure was performed by vascular technicians using the Accuson Sequoia Ultrasound Imager (Siemens Medical Solutions USA, Inc., Malvern, USA) at the University of Oklahoma Medical Center, Oklahoma City. The Sequoia uses 6L3 and 8L3 transducers that provide a linear array format with expanded MultiHertz^™^ multiple frequency imaging.

The FDA-approved CIMT test is done by rubbing the ultrasound probe (transponder) over each side of the subject’s neck to obtain measurements of the thickness of the common carotid artery walls. It is also possible to obtain information regarding the presence of occlusion from these scans, but the instrument does not distinguish between hard and soft plaques. The CIMT values correlate well with coronary artery findings (96% correlation) (27–29).

The IMT of the common carotid artery was measured in millimeters (15). The study group was provided with a duplex carotid screen (both arteries) using Doppler sonography. The distance between two echogenic lines corresponding to the lumen–intima interface and the media–adventitia interface of the carotid arterial wall visualized on B-mode vascular ultrasound gives the observed CIMT values (29, 30). The atherosclerotic CIMT score is expressed as a sum of the values determined in both arteries. Only subjects with a value entry in the ultrasound test were taken into account for statistical analysis (15).

### Anti-LPL, antioxidized LDL, or anti-LDL ELISA

The assays were performed as in the previous study (15). Lipoprotein lipase, oxidized LDL, or LDL was coated onto ELISA plates, and blocked with milk, and subject sera were added at 100-fold dilution and incubated overnight. The plates were washed and incubated with an antihuman IgG alkaline phosphatase conjugate, followed by a substrate and O.D. read at 405 nm.

### Lipid determination and high-density lipoprotein/LDL isolation

Cholesterol and triglyceride determination, and high-density lipoprotein (HDL)/LDL isolation were performed as described (15).

### Statistical analysis

Values are presented as mean ± standard deviation (SD). Statistical analysis was carried out using Student’s *t*-test, with *p* < 0.05 considered statistically significant.

## Results

**FIGURE 1:**
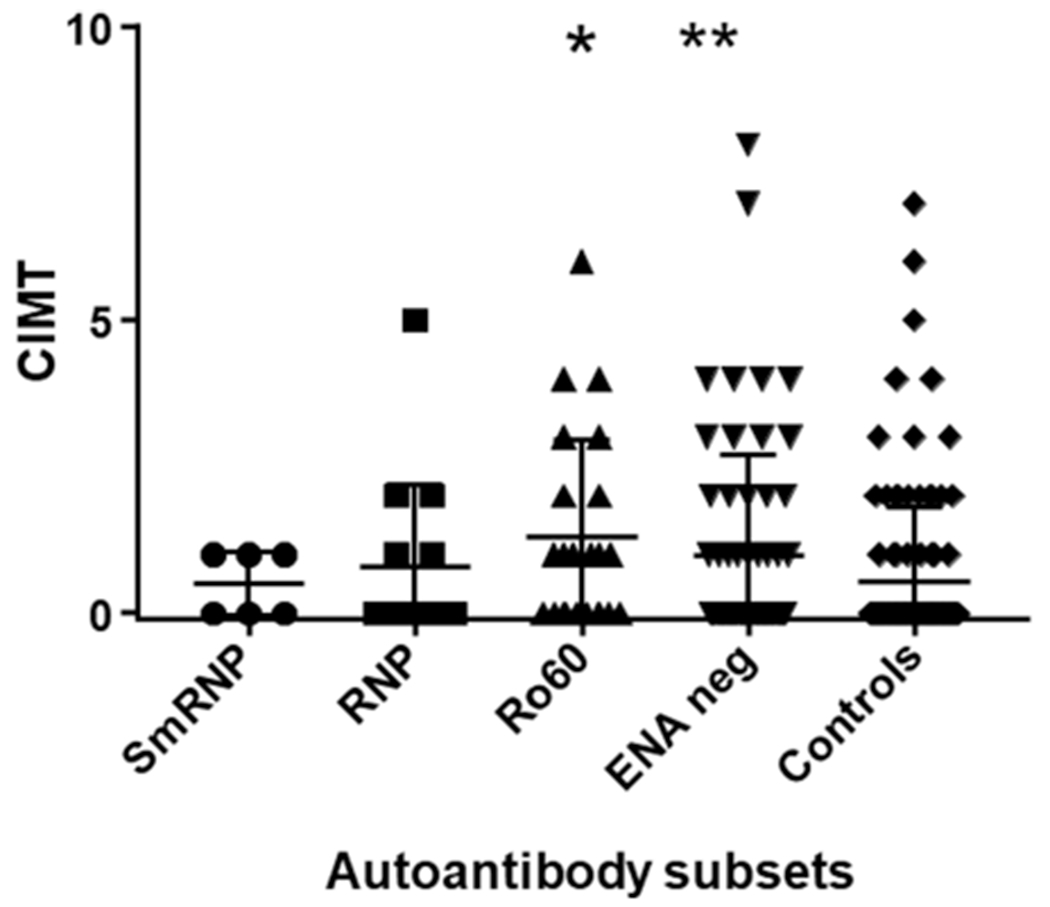
Carotid intima–media thickening (CIMT) in SLE autoantibody subsets and normal controls. CIMT was used to determine intimal thickening scores expressed on a scale of 0 to 10. SLE subjects were divided into subsets based on their autoantibody profile as determined by immunodiffusion studies and analyzed for CIMT scores. CIMT is given in millimeters. *p < 0.01 compared with control. **p < 0.05 compared with control.

**FIGURE 2:**
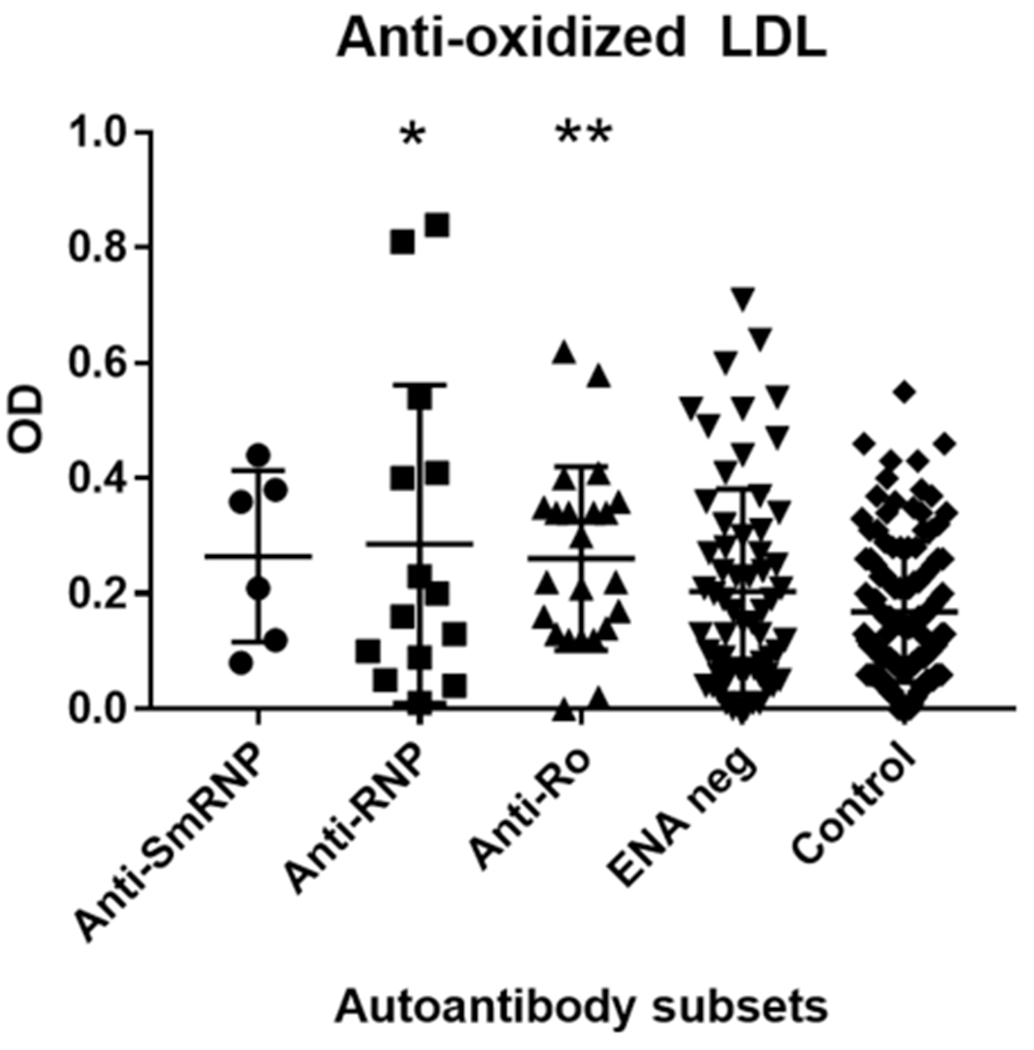
Antioxidized LDL antibodies in SLE autoantibody subsets and normal controls. Antibodies against oxidized LDL were determined as mentioned in “Subjects and methods.” SLE subjects were divided into subsets based on their autoantibody profile determined by immunodiffusion studies and analyzed for antioxidized LDL antibodies. *p < 0.005 compared with control. **p < 0.002 compared with control.

Double immunodiffusion studies showed that 63 SLE subjects (55.26%) did not have autoantibodies against extractable nuclear antigen (anti-ENA), 14 (12.2%) had antibodies against ribonucleoprotein (anti-RNP), 23 (20.18%) had anti-Ro60, 6 (5.26%) had anti-SmRNP, four (3.5%) had unidentified precipitin lines, and four (3.5%) had miscellaneous antibodies. CIMT scores were highest in the SLE subset with anti-Ro60 autoantibodies compared with all other lupus subsets and normal controls (Table 1, Figure 1). While elevated compared with other SLE subsets, the CIMT values were not statistically different. However, CIMT in this subset was statistically different from CIMT in the normal controls (*p* < 0.01) (Table 1, Figure 1). Antibodies against oxidized LDL were also elevated in this subset compared with other SLE subsets. However, antioxidized LDL antibodies in this subset were significant only when compared with normal controls (*p* < 0.002) (Table 1, Figure 2).

**FIGURE 3:**
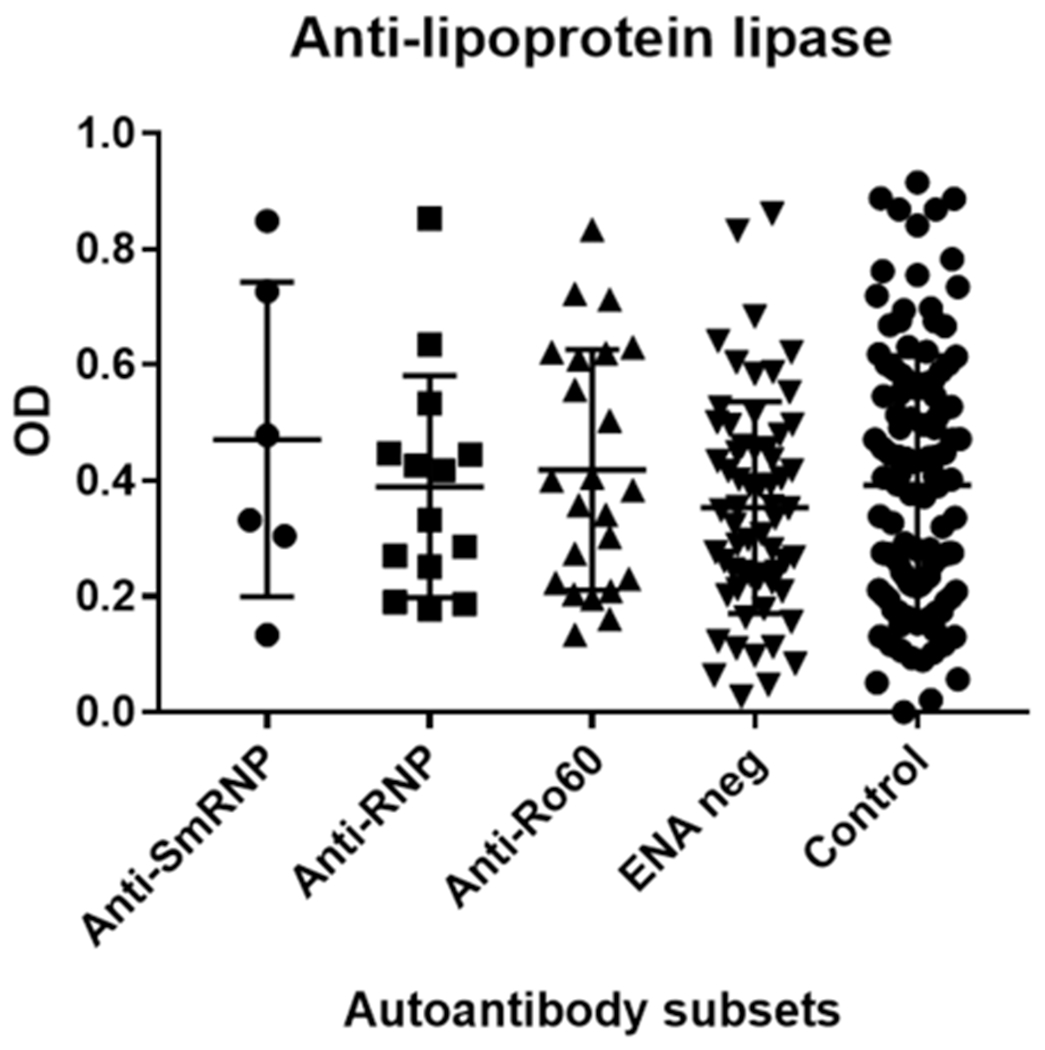
Antilipoprotein lipase antibodies in SLE autoantibody subsets and normal controls. Antibodies against antilipoprotein lipase were determined as mentioned in “Subjects and methods.” SLE subjects were divided into subsets based on their autoantibody profile determined by immunodiffusion studies and analyzed for antilipoprotein lipase antibodies. OD, optical density.

CIMT was significantly elevated in the SLE subset with autoantibodies against ENA compared with normal controls (*p* < 0.05). There were no differences in the levels of antibodies against oxidized LDL, LPL, and LDL in this and other subsets of SLE, nor in normal controls (Tables 1, 2, Figures 1–3).

The SLE subset with anti-RNP antibodies had significantly higher levels of antioxidized LDL antibodies (*p* < 0.005) compared with the control group but not within other SLE subsets (Table 1, Figure 2). CIMT and antibodies against LPL or LDL were not different in this subset compared with the control group and other SLE subsets (Table 1, Figure 1). The behavior of the anti-SmRNP SLE subset was not significantly different from that of normal controls and other SLE subsets with respect to CIMT in addition to antibodies directed against oxidized LDL, LDL, or LPL (Table 1, Figures 1–3). HDL-cholesterol, LDL-cholesterol, and triglyceride levels were not statistically different within the various subsets of SLE (Table 2).

There was no significant difference in CIMT when antioxLDL-positive/ALPL-positive or antioxLDL-negative/ALPL-negative SLE subjects were compared with either antioxLDL-positive/ALPL-positive or antioxLDL-negative/ALPL-negative normal controls.

Of the 23 subjects with anti-Ro60 autoantibodies, three had an unidentified line in the double immunodiffusion assay, while seven others had anti-La antibodies. One out of 14 subjects with anti-RNP also had an unidentified line in the double immunodiffusion assay. Therefore, in terms of the analysis carried out in this study, none of the antibodies reported here were counted multiple times in the CIMT analysis.

The observation that (a) antioxidized LDL and CIMT are significantly increased in SLE subjects with anti-Ro60 autoantibodies and (b) CIMT is significantly elevated in ENA-negative SLE subjects compared with normal controls appears to be the most interesting result obtained from this study.

## Discussion

Studies have shown that SLE subjects have a nine- to 50-fold risk for myocardial infarction compared with the general population (31, 32). While traditional risk factors contribute to CVD in SLE, non-traditional risk factors play an important part (33, 34). Contrary to what is seen in the general population, young subjects with premenopausal lupus have more commonly premature CVD (1). Atherosclerosis in SLE is linked to inflammation (35). SLE subjects suffering from a cardiovascular event are more likely to be diagnosed with lupus at an older age, to have a longer duration of lupus, to have a longer period of corticosteroid use, to be hypercholesterolemic, and to be postmenopausal than SLE subjects without any cardiovascular event (31, 33).

That immune dysregulation typical of lupus is important for CIMT progression and vascular complications is borne out by the observation that a higher damage index score and less aggressive immunosuppression are associated with an increased CVD (36–38). Both the innate and adaptive immune systems that trigger the inflammatory state of lupus may also be associated with the development and progression of CVD (39). The results of two earlier studies from our group and the data from this study support an important role for autoantibodies as a potentially increased risk for atherosclerosis in SLE.

Our study looked at the association of autoantibodies targeting RNP, SmRNP, Ro, La, oxidized LDL, LDL, or lipoprotein lipase with carotid intima–media thickening in SLE and control subjects. We found that the level of CIMT was the highest in the group with anti-Ro. This group also had statistically significant high levels of antibodies targeting oxidized LDL.

Studies have shown that the measurement of CIMT by ultrasound of the carotid arteries is a useful predictor of coronary artery disease associated with coronary disease events such as angina and myocardial infarction (22, 40). Carotid Doppler ultrasonography of the carotid artery, which is widely used to measure the intima–media thickness, has served well as a biomarker for atherosclerosis and plaque characterization (41). CIMT, a well-validated index for the detection of early-stage atherosclerosis, is associated with CVD, cerebrovascular disease, and peripheral vascular disease (42, 43). An atherosclerotic plaque is defined as either a focal wall thickening at least 50% larger than the surrounding vessel wall or a distinct intimal thickening of more than 1 mm that protrudes into the lumen and is separate from the adjacent boundary (44).

Carotid artery atherosclerosis is assumed to progress below the intimal layer in the subintima. In contrast, carotid plaque originates in the intimal layer and embodies the atherosclerotic process itself (45–47). A CIMT value of more than 1.5 mm or a focal intimal medial thickening of more than 50% of the surrounding area is a frequently reported threshold value to define diffuse plaque (48, 49). However, confusion arises as ultrasound resolution currently permits the visualization of discrete protuberant plaque lesions smaller than this threshold value. Also, different studies have even reported varying CIMT thresholds for plaque. One study defined plaque as focal thickening of the intima-media larger than 1 mm that was at least twice as thick as the neighboring normal CIMT, thus giving different definitions of plaque ranging from 0.5 to >1.5 mm (50). A different study then defined plaque as CIMT >1.2 mm (51). The European Mannheim consensus, however, defined plaque as a focal thickening that intrudes into the lumen by 0.5 mm or by 50% of the neighboring intimal–medial thickness or where CIMT is greater than 1.5 mm (52).

We know that patients with anti-Ro and anti-La have highly statistically significant elevations of antioxidized LDL and antiphospholipid antibodies (26). We studied antioxLDL antibodies in an SLE patient over a period of 137 months. We found that the level of antioxLDL was very high when we began studying this SLE patient, and it stayed highly elevated for almost the entire study period (23). We also found that rabbits immunized with Ro60 or Ro peptides develop an SLE-like disease with high levels of anti-Ro60 and intermolecular epitope spreading to La, oxidized LDL, and phospholipids (manuscript in preparation). However, the exact mechanism by which CIMT and antibodies against oxidized LDL arise in SLE subjects with anti-Ro is not known.

Ultraviolet irradiation or caloric stress may induce an increased expression of autoantigens. The ultraviolet-irradiated skin is better targeted by anti-Ro antibodies under experimental conditions. This manifestation has been appreciated in the skin of subjects with subacute cutaneous lupus erythematosus. In these subjects, the availability of autoantigens increases after sun exposure and such a factor can induce *in situ* formation of the Ro/anti-Ro immune complex (53). Ultraviolet exposure has been shown to increase free radical release by skin cells (54). Increased production of reactive oxygen species by this process or by the depletion of antioxidants or antioxidant enzymes by autoantibodies (55, 56) may increase oxidative stress, which may lead to LDL oxidation. Antilipoprotein lipase autoantibodies found in SLE are believed to allow LDL to persist in the circulation by hindering lipid transport further downstream (15). Therefore, LDL becomes an ideal candidate for oxidative modification. LDL modified in this fashion behaves like a neoantigen, allowing the host to see it as a non-self-antigen and inducing the host to make antibodies against such antigens (57, 58). Free radicals and antibodies to oxidized LDL have been implicated in the atherosclerosis found in SLE (59).

Oxidized LDL has been shown to be complex with plasma β2-glycoprotein I (β2GPI) and become autoantigenic, eliciting the production of specific antiphospholipid antibodies (60). We recently reported significantly elevated IgG antiphospholipid levels that correlated with antioxidized LDL in SLE (26). In an interesting twist to the investigative process, the question now being asked is whether atherosclerosis is an autoimmune disease. Elevated levels of oxLDL/β2GPI were first observed in SLE and antiphospholipid syndrome, and subsequently in coronary heart disease and type 2 diabetes mellitus. When subjects with chronic coronary heart disease were studied prospectively over a 2-year period, the early plasma concentrations of oxLDL/β2GPI were found to correlate with the number and severity of cardiovascular events (60). Recent work shows an association between anti-Ro and low IgM antiphosphoryl choline antibodies in SLE patients. Low IgM antiphosphoryl choline is associated with CVD in subjects who do not have antiphospholipid antibodies (61).

The associations of antioxLDL with the anti-Ro60, anti-RNP, or anti-SmRNP subsets did not show significant differences. Likewise, the association between anti-LPL and the various autoantibody subsets was not significant. It is difficult to determine whether this is a truly negative association caused by the relatively small sample size of the anti-Ro60, anti-RNP, or anti-SmRNP cohorts. However, we are confident that there is a real negative association of antioxLDL or anti-LPL with the anti-ENA subsets because of the sample size of this subset.

We acknowledge several limitations of this study, such as the absence of data on traditional risk factors such as smoking, arterial hypertension, and obesity, in addition to organ lesions such as lupus nephritis.

It would be of great interest to investigate whether subjects with subacute cutaneous lupus erythematosus have increased CIMT and elevated levels of antibodies against oxidized LDL and lipoprotein lipase since they do not have much systemic inflammation. Furthermore, exploring the relationship between autoantibody subsets in SLE and cardiovascular events would be extremely interesting. It is possible that cardiovascular events are augmented in an SLE subset with anti-Ro and elevated levels of antioxidized LDL antibodies, which could have significant clinical consequences.

## Figures and Tables

**TABLE 1:** Antibodies to low-density lipoprotein (LDL), oxidized LDL (oxLDL) and lipoprotein lipase LPL and SLE disease activity index (SLEDAI), carotid intima–media thickening (CIMT) in subsets of lupus patients and controls.

Number	ENA antibody type	Anti-LDL	Anti-oxLDL	Anti-LPL	SLEDAI	CIMT (mm)	Age (years)
23	Anti-Ro60	0.105 ± 0.104	0.261 ± 0.159**	0.419 ± 0.207	17.609 ± 15.23	1.304 ± 1.66*	50.261 ± 14.37
14	Anti-RNP	0.14 ± 0.139	0.286 ± 0.276^‡^	0.39 ± 0.192	18.31 ± 12.21	0.786 ± 1.424	38 ± 11.6
6	Anti-SmRNP	0.138 ± 0.122	0.265 ± 0.149	0.471 ± 0.27	20.67 ± 0.43	0.5 ± 0.55	36.833 ± 16.043
63	ENA negative	0.104 ± 0.094	0.408 ± 1.622	0.353 ± 0.183	18.43 ± 12.01	0.98 ± 1.709^†^	45.984 ± 12.41
117	Normal controls	0.089 ± 0.097	0.165 ± 0.131	0.392 ± 0.224	—	0.538 ± 1.256	43.58 ± 13.35

Anti-LDL, anti-oxLDL, and anti-LPL values are expressed as optical density measured at 405 nm. Carotid intima–media thickness is expressed in millimeters. Anti-ENA, anti-Ro 60, anti-RNP, and anti-SmRNP were determined by immunodiffusion.

* *p* < 0.01 compared with control.

** *p* < 0.002 compared with control.

† *p* < 0.05 compared with control.

‡ *p* < 0.005 compared with control.

**TABLE 2:** Total cholesterol, total triglycerides, LDL cholesterol, and HDL cholesterol in subsets of lupus patients and normal controls.

#	ENA	Total cholesterol	Total tri glycerides	LDL cholesterol	HDL cholesterol
23	Anti-Ro60	179.92 ± 48.68	123.38 ± 72.3	101.54 ± 38.73	53.16 ± 17.46
14	Anti-RNP	176.04 ± 38.56	166.56 ± 99.05*	101.86 ± 99.52	47.72 ± 10.61
6	Anti-SmRNP	188.15 ± 52.42	136.55 ± 74.06	116.1 ± 37.33	45.68 ± 9.62
63	Negative	212.05 ± 56.04**	170.95 ± 111.54***	124.12 ± 40.26	55.16 ± 23.05
117	Normal controls	192.31 ± 39.48	117.89 ± 69.61	109.46 ± 29.91	55.34 ± 15.09

* *p* < 0.025 compared with control.

** *p* < 0.007 compared with control.

*** *p* < 0.0002 compared with control.

## Data Availability

The original contributions presented in the study are included in the article/Supplementary Materials, further inquiries can be directed to the corresponding author.
